# Development of Nanoemulsions for Topical Application of Mupirocin

**DOI:** 10.3390/pharmaceutics15020378

**Published:** 2023-01-22

**Authors:** Bahjat Alhasso, Muhammad Usman Ghori, Barbara R. Conway

**Affiliations:** 1 Department of Pharmacy, School of Applied Sciences, University of Huddersfield, Queensgate, Huddersfield HD1 3DH, UK; 2 Institute of Skin Integrity and Infection Prevention, University of Huddersfield, Queensgate, Huddersfield HD1 3DH, UK

**Keywords:** eucalyptus oil, eucalyptol, mupirocin, nanoemulsion, topical delivery, permeation

## Abstract

Mupirocin (MUP) is a topical antibacterial agent used to treat superficial skin infections but has limited application due to in vivo inactivation and plasma protein binding. A nanoemulsion formulation has the potential to enhance the delivery of mupirocin into the skin. MUP-loaded nanoemulsions were prepared using eucalyptus oil (EO) or eucalyptol (EU), Tween^®^ 80 (T80) and Span^®^ 80 (S80) as oil phase (O), surfactant (S) and cosurfactant (CoS). The nanoemulsions were characterised and their potential to enhance delivery was assessed using an in vitro skin model. Optimised nanoemulsion formulations were prepared based on EO (MUP-NE EO) and EU (MUP-NE EU) separately. MUP-NE EO had a smaller size with mean droplet diameter of 35.89 ± 0.68 nm and narrower particle size index (PDI) 0.10 ± 0.02 nm compared to MUP-NE EU. Both nanoemulsion formulations were stable at 25 °C for three months with the ability to enhance the transdermal permeation of MUP as compared to the control, Bactroban^®^ cream. Inclusion of EU led to a two-fold increase in permeation of MUP compared to the control, while EO increased the percentage by 48% compared to the control. Additionally, more MUP was detected in the skin after 8 h following MUP-NE EU application, although MUP deposition from MUP-NE EO was higher after 24 h. It may be possible, through choice of essential oil to design nanoformulations for both acute and prophylactic management of topical infections.

## 1. Introduction

**Figure 1:**
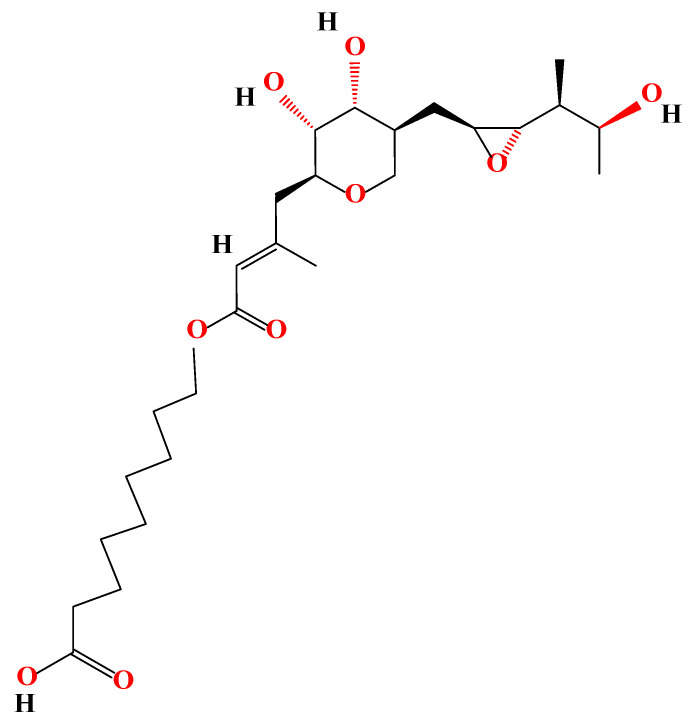
Chemical structure of MUP.

Mupirocin (MUP) is an effective antibacterial agent used widely in the treatment of superficial topical infections because of its broad-spectrum of activity [1] and antibiofilm characteristics [2]. MUP is an analogue of isoleucyl adenylate, created naturally by *Pseudomnas fluorescens* and known previously as pseudomonic acid A [3]. MUP is composed of a short chain fatty acid and monic acid as shown in Figure 1 [4]. 

It exerts its antibacterial activity by reversibly inhibiting bacterial isoleucyl-tRNA synthetase, leading to the inhibition of synthesis of bacterial protein [5]. This mode of action is unique among all antibacterial agents and is the likely reason there is no cross-resistance between MUP and other antibiotics [6]. The antibacterial activity of MUP ranges from bacteriostatic at low concentration to bactericidal at high concentrations [7] and is pH dependent, being higher at acidic pH [8]. 

The promising in vitro antibacterial activity of MUP against Gram-positive staphylococci, including methicillin-resistant *S. aureus,* (MRSA), the main causative microorganism of skin infections, drives its widespread use in prophylaxis and treatment of infections associated with skin, skin appendages and mucosal membranes [9]. In addition, MUP shows antibacterial activity against most streptococci with reported activity against some Gram-negative microorganisms such *as Haemophilus influenzae* and *Neisseria* spp. [7].

MUP is a white to off-white crystalline solid with a molecular weight of 500.6 g/mol [4]. It is very slightly water soluble (0.0265 g/L), maximal at pH 3.5–4.5 [4,7]. MUP is lipid soluble, with a Log P of 2.45 [10]. Its therapeutic efficacy is hindered by its short half-life (<30 min) and the emergence of resistance [8]. In addition, parenteral use is restricted due to metabolic inactivation and instability due to its high plasma protein binding affinity. Therefore, clinical applications are limited to topical skin infections and nasal carriage for *Staphylococcus aureus* decolonisation [7,11]. MUP has excellent potential as a topical antibacterial agent but clinical application is limited because of its poor permeability into/through the skin and lack of sustained delivery. Nanoformulations such as nanostructured lipid carriers [12], nanoliposomes and PEGylated nanoliposomes [7] can enhance target infected tissues and permeability into wounds and infected lesions, protect MUP in systemic circulation, improve burn healing and enhance topical antibiofilm activity. A nanoemulsion is an isotropic heterogenous system with transparent/translucent appearance. It is composed of two immiscible liquids (oil/water) with droplet size ranging from 20–400 nm stabilised by the aid of interfacial layer of surfactant. Three types of nanoemulsion are reported: oil-in-water, water-in-oil and bi-continuous nanoemulsion [13].

Nanoemulsions have several advantages over conventional formulations and even some advanced delivery systems such as microemulsions. These advantages include ultrafine droplet size with large surface area, high loading capacity and entrapment efficiency for lipophilic drugs, kinetic stability, capacity to solubilise both lipophilic and hydrophilic drugs, high permeation rate through the skin, extended release of drug and targeting the site of action [13,14]. They have been used for topical delivery of active substances including curcumin [15], naproxen [16] minoxidil [17] and tamoxifen [18]. 

Terpene essential oils have been widely reported as efficacious in the enhancement of transdermal drug permeation following incorporation into advanced pharmaceutical formulations such as nanoemulsions [19]. They disturb the stratum corneum lipid reversibly to enhance drug permeation and diffusivity through the skin [20]. In the current study, an *o*/*w* nanoemulsion was developed to deliver MUP topically using an essential oil as the oil phase and natural penetration enhancer. This dual approach may enhance the penetration and permeation of a drug into/through the skin and enhance antimicrobial activity. 

In this study, eucalyptus oil (EO), and its main constituent eucalyptol (EU), were used separately in the development of nanoemulsions. Their combination with MUP has been reported to demonstrate synergistic antimicrobial activity against a wide range of microorganisms [21]. However, oxidative decomposition of EU (the main component of EO) due to the exposure to air results in subsequent loss of antimicrobial activity [22]. Therefore, a nanoemulsion was considered as an effective formulation in encapsulating and protecting the EO and EU from hydrolysis and oxidation due to environmental impact [23]. 

As far as we know, this is the first study to report the formulation of a nanoemulsion of MUP using either EO or EU as an oil phase and stabilising the formulation by adding a mixture of surfactant and cosurfactant, Tween^®^ 80 (T80) and Span^®^ 80 (S80). The formulations were characterised, optimised and evaluated. The influence of the process parameters on the physicochemical properties of the nanoemulsions were studied. In addition, in vitro skin permeation was studied to determine the effect of the essential oil nanoemulsions on permeability of MUP compared to the current marketed cream formulation. 

## 2. Materials and Methods

### 2.1. Materials

MUP (purity > 98%) was purchased from Tokyo Chemical Industry UK Ltd. (Oxford, UK) and Discovery Fine Chemicals Ltd. (Leek, UK). Polyoxyethylene sorbitan monooleate (Tween^®^ 80), sorbitan mono oleic acid (Span^®^ 80), eucalyptol (purity 99%), eucalyptus oil (purity 100%) and absolute ethanol (purity ≥ 99.8) were all analytical grade and were purchased from Sigma Aldrich (Gillingham, UK). Acetonitrile (purity ≥ 99%), methanol (purity ≥ 99.5%) and ortho-phosphoric acid 85% were all HPLC grade and were purchased from Sigma Aldrich (UK).

Ultra-pure water was obtained from Barnstead Nanopure (Texas, TX, USA). Merck Strat-M^®^ membrane and B Braun^™^ hypodermic needle and adhesive tape (3M Transpore^®^) were purchased from Thermo Fisher Scientific (Warrington, UK). 

### 2.2. Solubility Determination

MUP solubility was determined in different components used in the formulation of nanoemulsions by adding an excess quantity into 2–3 mL of selected essential oil, surfactant, co-surfactant and deionised water separately in 10 mL stoppered glass centrifuge tubes. The centrifuge tubes were kept at 25 ± 1 °C in an isothermal water bath, Grant GLS Aqua 12 Plus (Grant Instruments Cambridge Ltd., Royston, UK), shaking at a rate of 100 rpm for 72 h in order to achieve equilibrium. After achieving equilibrium, the samples were removed from the water bath and centrifuged (Eppendorf Centrifuge 5702, Stevenage, UK) at 3000 rpm for 15 min. An aliquot was obtained from the supernatant of each sample and diluted with methanol (MeOH) prior to filtering through a 0.45 μm syringe filter. The filtered samples were analysed using HPLC in order to measure the amount of MUP dissolved in each solvent [24]. 

### 2.3. HPLC Method 

A reversed phase HPLC method was developed and validated using a Shimadzu HPLC, consisting of LC-10AT pump, LC-20AT autosampler and UV–Vis detector (SPD-20AV). A Waters XTerra MS C_18_ analytical column with 3.5 μm particle size, 150 mm length and 4.6 mm internal diameter was used with an isocratic mobile phase comprising phosphoric acid in water with pH (2.75 ± 0.05) (60%) and acetonitrile (40%). The mobile phase was run at a flow rate of 1 mL/min for 10 min with a volume of injection of 20 μL. The temperature was maintained at 40 °C and a wavelength of 220 nm was used for detection of MUP. The HPLC method was validated according to ICH Topic Q2 (R1) guidelines [25]. 

### 2.4. Gas Chromatography (GC) Method

A GC method was developed and validated according to ICH [25] to calculate the percentage of EU in EO of the various sources. Briefly, a 6890N Agilent GC system was used attached with a flame ionisation detector (FID). Helium (carrier gas) was used as the mobile phase at a flow rate of 1 mL/min. The LM-20 fused silica capillary column (30 m × 0.25 mm × 0.25 μm) was was set at an initial temperature of 60 °C and programmed to rise at 5 °C/min starting from 60 °C with a hold time of (5 min) to 160 °C with a hold time of (5 min). The temperature of the injector and detector was controlled at 220 °C and 250 °C, respectively, with an autosampler using 1 µL. The pressure was set at 4.3 bar and the run time was 25 min. 

### 2.5. Construction of Pseudoternary Phase Diagrams

**Figure 2:**
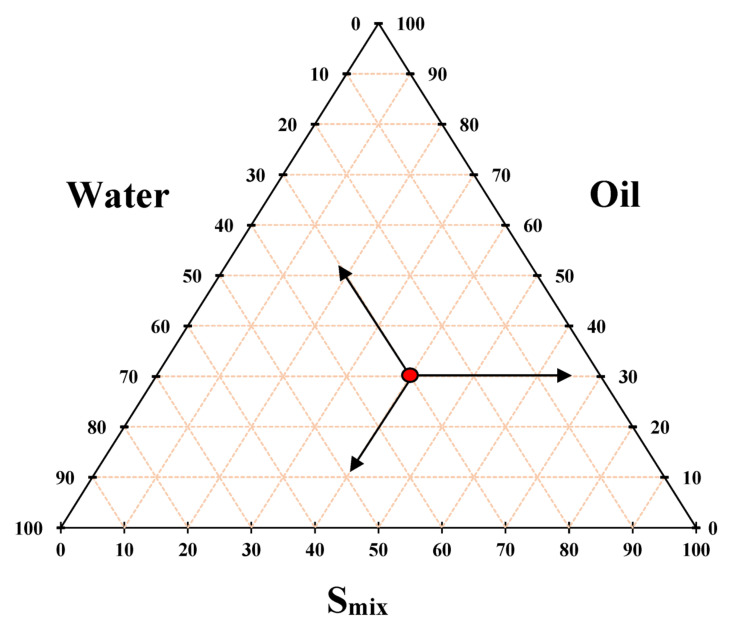
Typical pseudoternary phase diagram and scheme for determining titration points.

**Figure 3:**
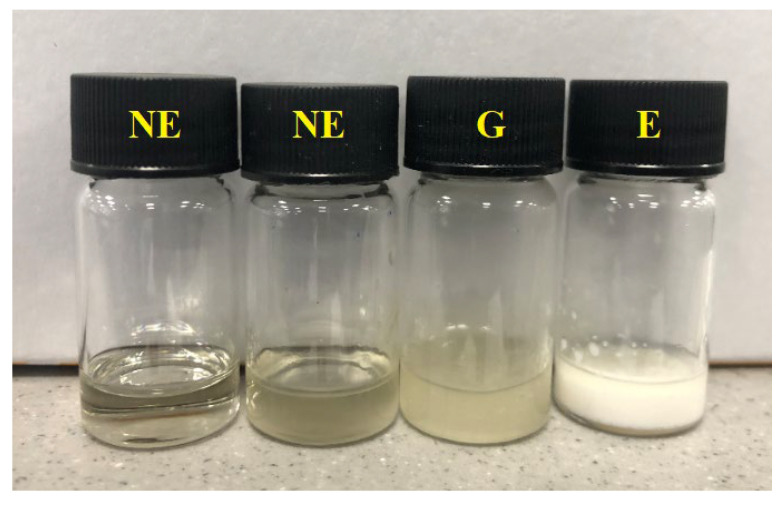
The visual determination of formulations in construction of pseudoternary phase diagram (NE, nanoemulsion; G, gel and E, emulsion).

The aqueous titration method was used to construct ternary phase diagrams [26]. The three components of the pseudoternary phase diagram comprising surfactant:co-surfactant mixture (S_mix_), oil and water are represented typically in Figure 2. The S_mix_ ratio was fixed at 1:1, 2:1, 3:1 and 4:1 based on a *w*/*w* ratio and the type of oil used. T80, S80 and selected essential oil were used as surfactant, co-surfactant and oil, respectively. The oil was mixed with S_mix_ in a series of ratios: 1:9, 2:8, 3:7, 4:6, 5:5, 6:4, 7:3, 8:2 and 9:1 (% *w*/*w*). Each mixture was then titrated with the addition of 100 μL of deionised water at room temperature (25 °C). The titrated deionised water, oil and S_mix_ were vortexed at 1500 rpm using a Stuart vortex mixer (Barloworld Scientific Limited, Stone, UK) at room temperature for 2 min, and then left for 15 min in order to equilibrate and were observed visually. Deionised water (100 μL) was added sequentially and three characteristics were reported following visual inspection. When the liquid was easily flowable, and either transparent or translucent, this was identified as a nanoemulsion, with both shown in Figure 3. The points were recorded and plotted on a triangular graph as a ternary phase diagram using MS Excel 2019. The regions where the liquid was easily flowable, cloudy or milky were classified as an emulsion. If there was a transparent or milky semisolid which did not change its meniscus upon tilting at a 90° angle, this was identified as a gel, nanoemulgel or emulgel, also shown in Figure 3. 

### 2.6. Preparation of Blank Nanoemulsions

The aqueous phase was prepared by heating Tween 80 and deionised water in at 60 °C on a hotplate stirrer (Stuart CD162, London, UK), mixing at 500 rpm for 10 min. The lipid phase was prepared by mixing a defined quantity of Span 80 and essential oil under the same conditions as shown in Table 1. The two phases were mixed by adding the aqueous phase to the lipid phase under vigorous stirring at 800 rpm and 60 °C for a 5 min on a hotplate stirrer. This coarse milky emulsion was homogenised at 25,000 rpm for 10 min using a high shear homogeniser (Ystral GmbH D-7801 Dottingen, X1020 homogeniser, Ballrechten-Dottingen, Germany) and ultrasonicated at 60% amplitude for 15 min using a probe ultrasonic homogeniser (Model 3000MP Ultrasonic homogeniser, Biologics Inc., Manassas, VA, USA) with cooling during the ultrasonication. The nanoemulsion was cooled to 25 °C prior to characterisation.

### 2.7. Optimisation of the Nanoemulsion Formulation 

#### Central Composite Design

After determining nanoemulsion regions, three-factor central composite design (CCD) was employed, using Minitab^®^18 software, to optimise nanoemulsion formulation. The design examined the effect of three independent formulation variables at three levels (low, medium and high): homogenisation time (5–10 min, *X*_1_), ultrasonication time (10–20 min, *X*_2_) and amplitude percent (50–70%, *X*_3_) on the dependent (response) variables: average particle size (*Y*_1_) and PDI (*Y*_2_) (Table 2). Fourteen experiments were run randomly in addition to the three central point replications as shown in Table 3. A mathematical formula for the response can be created as follows:

(1) $$Y_{i}=a0+a_{1}X_{1}+a_{2}X_{2}+a_{3}X_{3}+a_{12}X_{1}X_{2}+a_{13}X_{1}X_{3\;}+a_{23}X_{2}X_{3}+a_{11}X_{1}^{2}+a_{22}X_{2}^{2}+a_{33}X_{3}^{2}$$

where *Yi* is the predicted response, *Xi*, the independent variable, *a*0, a constant, *ai*, *aii* and *aij* the linear, quadratic, and interactive coefficients, respectively.

### 2.8. Preparation of MUP-Loaded Nanoemulsions

MUP nanoemulsions were prepared using the same method as Section 2.6, except MUP was incorporated into the lipid phase. MUP was dissolved in absolute ethanol and mixed with a defined quantity of selected essential oil as shown in Table 1 and the ethanol was removed by rotary evaporation. Fresh oil was added to compensate for any weight decreases. S80 was added to the mixture and mixed at 60 °C for 5 min. The aqueous phase was added to the oil phase and mixed vigorously at the same temperature using stirring at 800 rpm for 5 min. The resultant milky coarse emulsion was then homogenised and ultrasonicated under the same conditions described in Section 2.6.

### 2.9. Measurement of Size, Polydispersity Index and Zeta Potential of Nanoemulsions 

A nanoemulsion sample was diluted in ultrapure water at a ratio of 1:3. Then, the system was equilibrated for 60 s at 25 °C in order to determine the droplet size and polydispersity index (PDI) using Zetasizer (Malvern Nano ZS, Malvern, UK). Particle size was confirmed using nanoparticle tracking analysis (NTA, NanoSight NS300, Malvern Panalytical, Malvern, UK). The zeta potential was measured by diluting 10 μL of nanoemulsion sample in 990 μL of ultrapure water using a dip cell. The system was equilibrated for 120 s at 25 °C. All the measurements were carried out in triplicate and represented as mean (±SD).

### 2.10. Determination of pH 

A digital pH meter (VWR Symphony SB70P pH Meter, Lutterworth, UK) was used for the measurement of the pH of the nanoemulsions. All the measurements were carried out at room temperature (25 °C) and in triplicate and expressed as mean and standard deviation (mean ± SD). 

### 2.11. Determination of the Nanoemulsion Viscosity

A Bohlin Gemini cone and plate rheometer (Malvern Instruments Ltd., Malvern, UK) was used for the measurement of the viscosity of the nanoemulsion formulations. The measurement was carried out at room temperature (25 °C) using a shear ramp between 0.1 and 100 s^−1^. The measurements were carried out in triplicate and expressed as mean and standard deviation (mean ± SD). 

### 2.12. Determination of Entrapment Efficiency (EE%)

The amount of drug in the formulation was measured by solubilizing the nanocarrier in MeOH, filtering and analysing by HPLC. The percentage of drug content was calculated as encapsulation efficiency using Equation (2) [27,28]:

(2) $$\mathrm{Incorporation}\;\left(\mathrm{entrapment}\right)\;\mathrm{efficiency}\%=\frac{\mathrm{Drug}\;\mathrm{quantity}\;\mathrm{in}\;\mathrm{nanocarrier}\;\left(\mathrm{nanoemulsion}\right)}{\mathrm{Initial}\;\mathrm{drug}\;\mathrm{quantity}}\times 100\;$$

### 2.13. Fourier Transform Infrared (FTIR) Spectroscopy

A Nicolet IS 50 FTIR spectrometer equipped with an iD7 ATR accessory (Thermo Scientific Fisher, Waltham, MA, USA) was used to perform spectroscopic measurements with a background run used as a negative control. The spectra used ranged from 400 to 4000 cm^−1^ with a resolution of 4 cm^−1^ and 32 scans. The samples investigated by FTIR were a blank nanoemulsion of EO, blank nanoemulsion of EU, MUP dissolved in MeOH nanoemulsion of EO- and EU-loaded MUP. 

### 2.14. Thermodynamic Stability Study

Selected formulated nanoemulsions were subjected to both long-term and accelerated stability tests in order to determine stability.

#### 2.14.1. Long-Term Stability Studies

The selected formulations were stored for 3 months at 4 °C and 25 °C. The droplet size, PDI and zeta potential were measured in triplicate each month for three months and the mean values and SD were calculated.

#### 2.14.2. Accelerated Stability Studies

##### Heating–Cooling Cycle

This was investigated by placing the samples in the refrigerator at 4 °C and oven at 45 °C for 48 h at each temperature to complete one cycle. This stage comprised six cycles and samples which remained stable at these temperatures were progressed to the next step. The particle size, PDI and zeta potential of the stable samples were measured in triplicate and expressed as mean ± SD. The samples were inspected visually for any signs of sedimentation, creaming and coalescence.

##### Centrifugation

Samples considered stable after the heating–cooling cycle were then centrifuged at 3500 rpm for half an hour and visually inspected for any signs of sedimentation or creaming. The particle size, PDI and zeta potential of the stable samples were measured in triplicate and expressed as mean ± SD. Nanoemulsions considered stable were progressed to the next step.

##### Freeze–Thaw Cycle

The samples were frozen at −18 °C for 48 h and then thawed at 25 °C for next 48 h in order to complete one cycle. Three cycles were carried out for each sample. The particle size, PDI and zeta potential of the stable samples were measured in triplicate and expressed as mean ± SD. 

All three stages must be passed successfully in order to consider the sample as stable [24].

### 2.15. In Vitro Release and Skin Permeation Studies

#### 2.15.1. In Vitro Release Studies of Nanoemulsion

In vitro release studies were carried out using Franz diffusion cells. The effective diffusion was determined for a Strat-M membrane over a diffusion area of 2.5 cm^2^ and receiver chamber capacity of 15 mL. The temperature was maintained at 37 ± 1 °C throughout the experiment with the magnetic stirrer rotating at 100 rpm for 24 h. The receiver chamber was filled with methanolic phosphate buffered saline (MeOH-PBS) (5:5). The formulation (nanoemulsion and control) was placed in the donor chamber which was sealed with Parafilm^®^ in order to minimise evaporation. An aliquot (0.5 mL) was withdrawn from the receiver chamber and substituted with 0.5 mL of fresh medium of MeOH-PBS at a regular interval of one hour for 24 h. The withdrawn samples were analysed by HPLC without dilution. 

#### 2.15.2. Preparation of the Skin and In Vitro Skin Permeation Studies of Nanoemulsion

Full thickness porcine ear skin was acquired from a local abattoir and was used as fresh or kept frozen at −18 °C and used within one month. 

In vitro skin permeation studies were carried out using Franz diffusion cells (PermeGear, Inc., Hellertown, PA, USA). The effective diffusion was occurred through a diffusion area of 3.14 cm^2^ and receiver chamber capacity of 15 mL. The temperature was maintained at 37 ± 1 °C throughout the experiment. The receiver chamber was filled with MeOH-PBS (5:5). The formulation (nanoemulsion or Bactroban^®^ cream as a control) was placed in the donor chamber and sealed with Parafilm^®^. An aliquot (0.5 mL) was withdrawn from the receiver chamber and substituted with 0.5 mL of fresh medium of MeOH-PBS at a regular interval of one hour for 24 h. The withdrawn samples were analysed by HPLC. After 8 and 24 h, the experiment was stopped, and any residual formulation was removed from the skin. The treated skin was left for 20 min prior to tape stripping.

#### 2.15.3. Determination of MUP Deposited in Skin

The tape-stripping method was used to determine the amount of MUP localised in different layers of the skin. Coupling tape stripping with cyanoacrylate superglue application enables determination of the amount of the drug deposited in the appendages such as hair follicles. Dissection of the remaining skin into small pieces and homogenising it enables determination of drug remaining in the skin [29,30].

Tape stripping was carried out by applying defined size pieces (2.5 × 2.5 cm^2^) of adhesive tape (3M Transpore^®^) 15 times, and then placing the tapes in 5 mL of MeOH. The tapes were divided into 4 groups as follows: group 1 (tape 1), group 2 (tape 2–5), group 3 (tape 6–10) and group 4 (tape 11–15). The second step involved the combination of cyanoacrylate with adhesive tape step. It was carried out by applying a small amount of cyanoacrylate on the stripped skin and leaving it for 10 min. After that, it was striped twice, and the tapes were immersed in 5 mL of MeOH (group 5). The final step was cutting the whole skin into small pieces and placing them in 5 mL ofMeOH, and this was designated as group 6. All the tubes (group 1–6) were sonicated for 30 min and then centrifuged at 400 rpm for 20 min. After that, the supernatant was analysed by HPLC after filtering (0.22 µm). This process was used to quantify the amount of MUP deposited in the skin. 

### 2.16. Statistical Data Analysis

All the results and responses in this study were calculated and measured in triplicate and were expressed as mean ± SD. Analysis of variance (ANOVA) was used to test all mean values using MS Excel 2019. The differences were considered as statistically significant if the *p* value was less than 0.05. 

## 3. Results and Discussion

### 3.1. Solubility of MUP

Although MUP was more soluble in EO (21.00 ± 0.17 mg/mL) than EU (7.93 ± 0.252 mg/mL) (Table 4), this was not enough to formulate a nanoemulsion with a therapeutic dose, i.e., 2% as in the marketed product. Therefore, solubility was increased by dissolving it in an organic solvent and then evaporating the solvent under a reduced pressure (vacuum). In order to carry out this step, several organic solvents were screened including acetonitrile, methanol and ethanol. MUP solubility was 189.66 ± 0.52 mg/mL, 179.24 ± 0.34 mg/mL and 32.86 ± 0.21 mg/mL in methanol, ethanol and acetonitrile, respectively. However, the comparative tolerability of ethanol made it more suitable and appropriate to be used in this formulation [31]. The low solubility of MUP in deionised water is due to its lipophilicity; however, its solubility increased in aqueous T80 solution (4.9% *w*/*w*). A similar effect of T80 on nimesulide and spironolactone solubility was reported by [32] and [33]. On the other hand, the solubility of MUP was increased slightly when EO was mixed with S80 and three-fold when S80 was mixed with EU. In addition, the incorporation of Tween80 and Span80 with EO and EU separately increased the solubility of MUP in both oils compared with the oils used alone. The synergistic effect of the surfactant:cosurfactant mixture increased the solubility of MUP two-fold and three-fold compared to the solubility of MUP in each oil alone. This is similar to findings reported by [34]. 

These studies informed the selection of the medium used in the receiver chamber in the Franz diffusion cell. In order to achieve sink conditions, the solubility of the lipophilic drug (MUP) in the dissolution medium should be at least three times higher than the saturated concentration of drug (amount of drug in dosage form). Therefore, the solubility of MUP was examined using different solvents. Phosphate buffered saline pH 7.2–7.6 and MeOH-PBS with three different ratios (1:9, 3:7 and 5:5) were used to test solubility of MUP. It was found that MUP was most soluble in MeOH-PBS pH 7.2–7.6 (5:5) so this was used as the receiver medium. 

### 3.2. HPLC Method Development and Validation

MUP was detected at wavelength 220 nm with a mean retention time of 4.18 min. No peak was identified at the retention time of MUP due to other components of the nanoemulsions. The percentage relative standard deviation of R^2^ and slope were less than 1% (0 and 0.89%, respectively). These results confirmed the linearity and reproducibility of the HPLC method and the results agreed with the criteria proposed by [35]. Furthermore, the LOD and LOQ were calculated as 1 and 3.04 μg/mL, respectively.

### 3.3. GC Method Development and Validation

The GC chromatogram showed the peak of EU at a retention time of approximately 19.94 min and blank solvent (methanol) at approximately 6.30 min. In addition, the percentage of relative standard deviation of R^2^ and slope was calculated as 0.07 and 1.15, respectively. The LOD and LOQ were calculated from the calibration curve and found to be 83.38 μg/mL and 252.68 μg/mL, respectively.

### 3.4. Construction of Peudoternary Phase Diagrams

**Figure 4:**
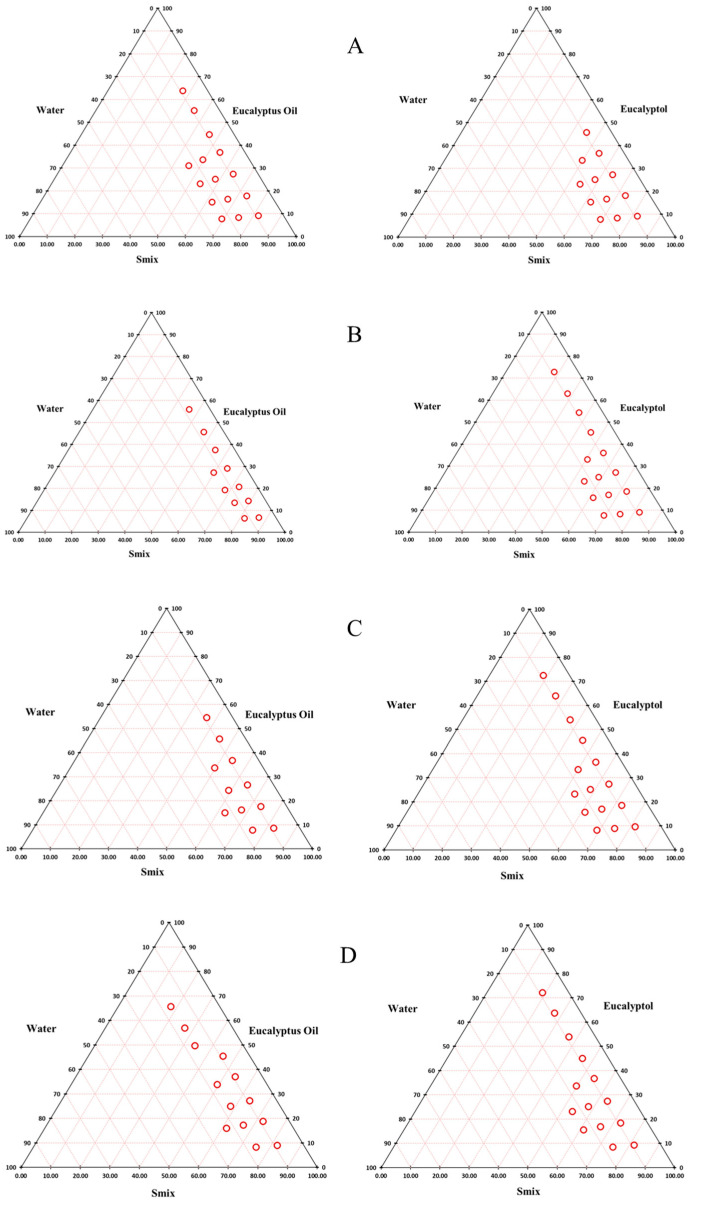
Pseudoternary phase diagrams of EO and EO with water and different ratios of surfactant mixture (T80:S80): (**A**) S_mix_ 1:1, (**B**) S_mix_ 2:1, (**C**) S_mix_ 3:1 and (**D**) S_mix_ 4:1. Circles indicate areas of nanoemulsion formation.

Nanoemulsions were formed in oil-rich regions in the border areas of the diagram where there are low water and oil ratio and high S_mix_ ratios (Figure 3). All the pseudoternary phase diagrams in Figure 4 exhibited similar regions for nanoemulsions. The formation of *o*/*w* or *w*/*o* nanoemulsion was based on the physicochemical properties of the surfactant including chemical structure and its solubility in water and oil [36]. In addition, the size of *w*/*o* nanoemulsion region decreased as the hydrophile/lipophile balance (HLB) value increased (due to the increase in surfactant concentration). HLB theory states that the type of surfactant tends to produce the type of emulsion in which it is more soluble in the dispersion (external) phase, i.e., the S_mix_ (T80:S80, 1:1) with HLB value 9.65 tends to produce a *w*/*o* emulsion or nanoemulsion [37]. An increase in the Tween 80 content of S_mix_ resulted in increasing HLB values, e.g., HLB value of S_mix_ 2:1, 3:1 and 4:1 is 11.576, 12.325 and 12.86, respectively. Any increase in tT80 and, therefore S_mix_ ratio, results in a phase shift from *w*/*o* to *o*/*w* type. In addition, the size of *w*/*o* nanoemulsion region decreased, forming a larger *o*/*w* nanoemulsion region and vice versa, according to HLB theory [38], Figure 4.

Figure 4B,C show the contrasting behaviour of EO and EU. EU tends to form more stable *o*/*w* nanoemulsions than EO at the same ratio of T80 and S80 based on the required HLB for each oil. This study is useful to determine the ability of each oil to form a stable emulsion in combination with the appropriate surfactant and cosurfactant. 

### 3.5. Preparation and Optimisation of Nanoemulsions

#### 3.5.1. Optimisation of Nanoemulsion

Nanoemulsion formulation and optimisation was carried out by CCD and responses for the various nanoemulsions were recorded and tabulated as shown in Table S1 (Supplementary Materials).

##### Dependent Variable 1: Droplet Size

Minitab software proposed a quadratic response surface model with the significance (*p* value) less than 0.05. The model was accepted with results indicating that the lack-of-fit is statistically non-significant. Although the R squared value for droplet size was slightly low (0.8922), the actual and predicted values of droplet size are in good agreement as shown in Table S1 (Supplementary Materials).

ANOVA data shown in Table S2 (Supplementary Materials) confirm the suitability and statistically significance of this model for droplet size. The data show that the impact of the three independent variables (homogenisation time, ultrasonication time and amplitude percent) on droplet size of nanoemulsion are statistically significant (*p* < 0.05). The quadratic equation generated by Minitab software for droplet size shown in Equation (3)

Size (*Y_1_*) = 157.7 − 1.99 *X_1_* − 3.10 *X_2_* − 1.158 *X_3_* − 0.024 *X_1_^2^* + 0.0687 *X_2_^2^* + 0.00670 *X_3_^2^* + 0.0367 *X_1_X_2_* + 0.0196 *X_1_X_3_* + 0.00719 *X_2_X_3_* (3)

**Figure 5:**
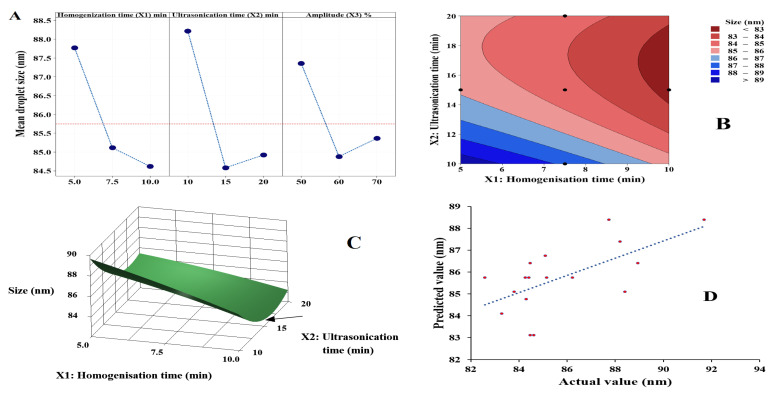
Results for data analysis of droplet size of nanoemulsion: (**A**) main effect plot, (**B**) contour plot, (**C**) response surface plot and (**D**) actual versus predicted plot.

Based on this equation, the effect of independent variables on droplet size were homogenisation time > ultrasonication time > amplitude percent (Figure 5A). The significant effect of *X_1_*, *X_2_* and *X_3_* might be the reason for the fitting of the droplet size data to the quadratic model. 

Additional data provided in Figure 5B indicate that an increase in *X_1_* is followed by a reduction in nanoemulsion droplet size, in line with the assumption derived from the quadratic equation of size. While the increase in *X_2_* and *X_3_* resulted in the reduction in the droplet size until reaching a mid-point, an increase resulted in a negative effect (size enlargement) as shown in Figure 5A,C. The relationship between the actual and predicted values of droplet size are shown in Figure 5D indicating that the model has demonstrated good power of prediction. 

##### Dependent Variable 2: PDI

The software suggested the use of a three-factor interaction with a *p* value less than 0.05 as a statistically significant indicator. The model was accepted with lack-of-fit being statistically non-significant. Although the R squared value for droplet size was very low (0.6758), the actual and predicted values of droplet size were fitted in good agreement (Table S1, Supplementary Materials).

ANOVA (Table S3, Supplementary Materials) confirmed a statistically significant influence of ultrasonication time on PDI while homogenisation time and amplitude percent did not have a significant effect on PDI. 

**Figure 6:**
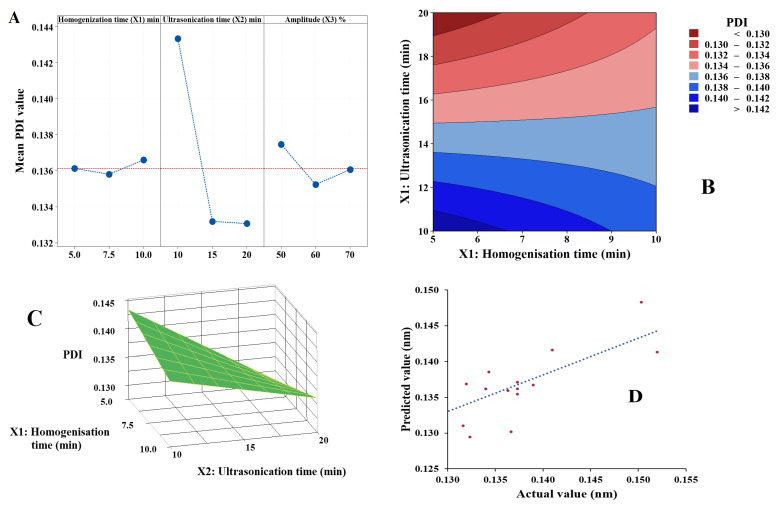
Results for data analysis of PDI of nanoemulsion: (**A**) main effect plot, (**B**) contour plot, (**C**) response surface plot and (**D**) actual versus predicted plot.

The software was used to generate a polynomial equation as shown in Equation (4). Results indicated a statistically significant effect of ultrasonication time (*X_2_*) on the PDI of nanoemulsion which agreed with the results presented in Figure 6A.

PDI (*Y_2_*) = 0.2514 − 0.00566 *X_1_* − 0.00600 *X_2_* − 0.001320 *X_3_* + 0.000190 *X_1_X_2_* + 0.000048 *X_1_X_3_* + 0.000059 *X_2_X_3_* (4)

The increase in the ultrasonication time (*X_2_*) accompanied by the reduction in the PDI of the nanoemulsion is shown in Figure 6B, supporting the assumption of the polynomial equation. and confirming the individual effect of each independent variable. So, increasing the time of ultrasonication decreases PDI, while increasing the homogenisation time increases PDI. Additionally, increasing amplitude percent had a similar impact as homogenisation time (Figure 6A,C). Figure 6D indicates a linear relationship between the predicted and actual values of PDI, conforming a good fit for this predictive model. 

**Figure 7:**
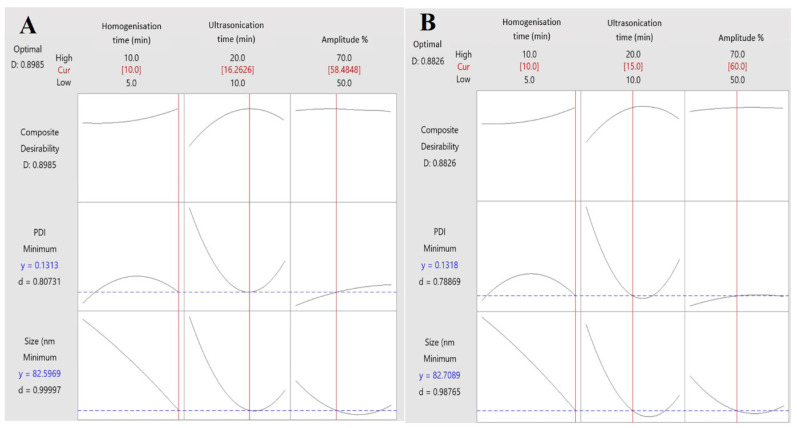
Numerical response optimiser data for nanoemulsion by CCD (**A**) the suggested optimised formulation and (**B**) the actual optimised formulation.

Figure 7 shows the optimised parameters with the predicted results. The minimal droplet size and PDI as the optimised goal. The obtained desirability was 0.8985 for the suggested parameters: homogenisation time, 10 min; ultrasonication time, 16.26 min and amplitude, 58.48%. The predicted droplet size and PDI for the suggested formulation were 82.59 nm and 0.131, respectively, as shown in Figure 7A. The actual optimised formulation had a similar desirability value (0.8826) for the used parameters. In addition, the optimised formulation in this study was obtained by setting homogenisation time as 10 min, ultrasonication time as 15 min and 60% for amplitude. The optimised parameters resulted in formulations with 82.71 nm and 0.132 as droplet size and PDI, respectively, as shown in Figure 7B. 

#### 3.5.2. Influence of Homogenisation Time 

**Figure 8:**
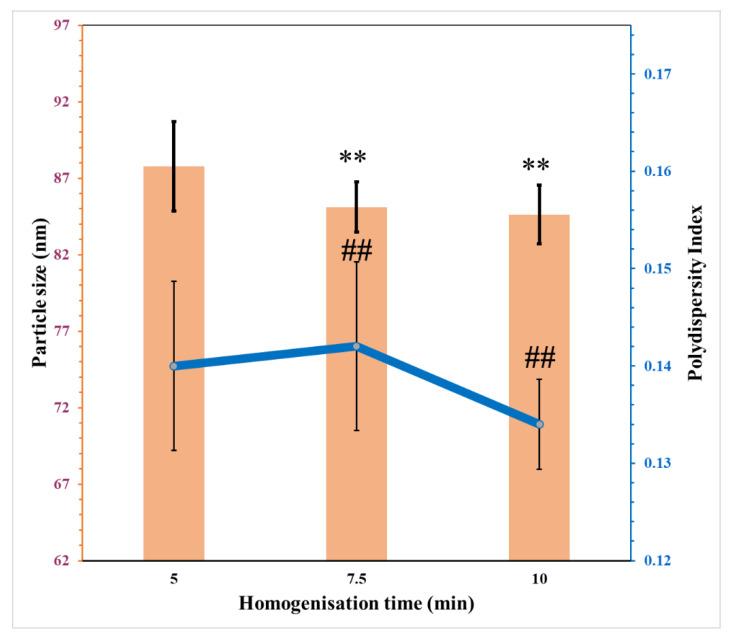
The effects of homogenisation time on the particle size and PDI of nanoemulsion (mean ± SD, n = 3). ** not significant compared to particle size of nanoemulsion homogenised for 5 min; ## not significant compared to PDI of nanoemulsion homogenised for 5 min.

Figure 8 shows the reduction in the average particle size with increasing duration of homogenisation at a constant speed. This is similar to previous findings [39]. However, this was not the case for PDI as increasing homogenisation time from 5 min to 7.5 min resulted in a slight increase in the PDI value. This is due to the formation of droplets with non-uniform shapes with a wide distribution of droplet sizes [40]. Extending the duration further decreased the PDI. Affandi et al., 2011 [40] reported a similar issue in their work on optimisation of astaxanthin nanoemulsion formulations. Therefore, a homogenisation duration of 10 min was adopted.

#### 3.5.3. Influence of Ultrasonication Time

**Figure 9:**
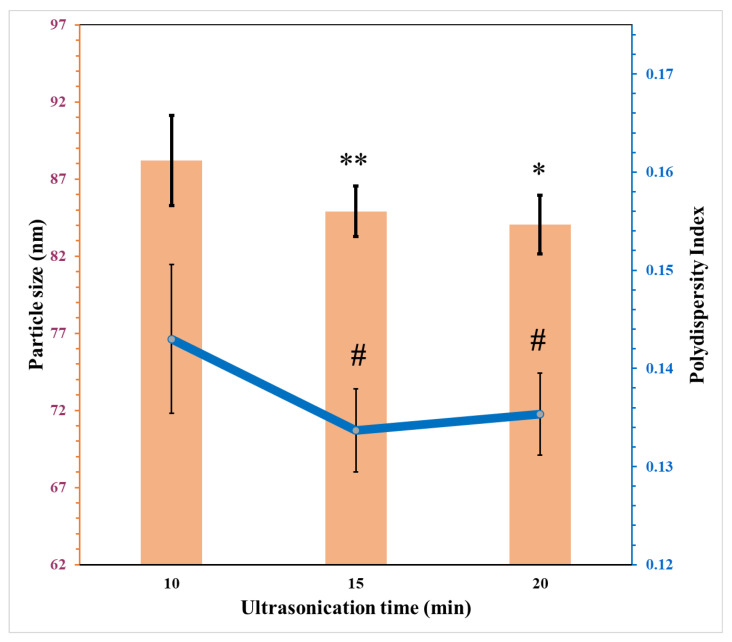
The effects of ultrasonication time on the particle size and PDI of nanoemulsions (mean ± SD, n = 3). * significant compared to particle size of nanoemulsion ultrasonicated 10 min; ** not significant compared to particle size of nanoemulsion ultrasonicated for 10 min; # significant compared to PDI of nanoemulsion ultrasonicated for 10 min.

The effects of the time for ultrasonication on the average particle size and PDI are shown in Figure 9. There is a linear relationship between the increase in the duration of ultrasonication and the reduction in the average particle size but not PDI. This was expected and described in previous studies [41,42,43,44,45]. The formation of a nanoemulsion differs from a coarse emulsion in the need to apply high external stress (shear rate). This helps in deformation of the droplets and results in smaller droplets. In other words, there is a need to increase Weber number, r, to deform droplets and reduce the size as described in Equation (5):

(5) $$We=\frac{G\eta r}{2\gamma }$$

where: 

*We* is Weber number which express droplet deformation.

*G* is the gradient of velocity 

*η* is the viscosity.

*r* is droplet radius

γ is the interfacial tension

The breakup of droplets depends on the type and the amount of applied stress to the droplets in addition to the ability of the droplets to resist that deformation, i.e., Laplace pressure.

Despite the results showing a minimum size at time 20 min, PDI started to increase very slightly beyond 15 min of ultrasonication. This might be attributed to the coalescence of droplets. The surfactant’s ability to be adsorbed onto the newly formed droplets determines the coalescence rate of droplets. Therefore, higher concentrations of surfactants are needed to control the surface activity and stabilise the formulation [42]. In addition, the higher ultrasonic temperature has unwanted complex effects on the emulsifying properties of the surfactant. Increases in irradiation power result in an increase in the rate of energy distribution within the system which in turn elevates the temperature. This elevated temperature causes coalescence, resulting in irregular increases in the size of droplets within the system [46]. It also reduces the viscosity of emulsion, interfacial tension and Laplace pressure. All these factors can disrupt the droplets, enhance the rate of coalescence and increase droplet size [46]. Therefore, the selected ultrasonic time was limited to 15 min for nanoemulsion formulations in this study. 

#### 3.5.4. Influence of Ultrasonic Amplitude

**Figure 10:**
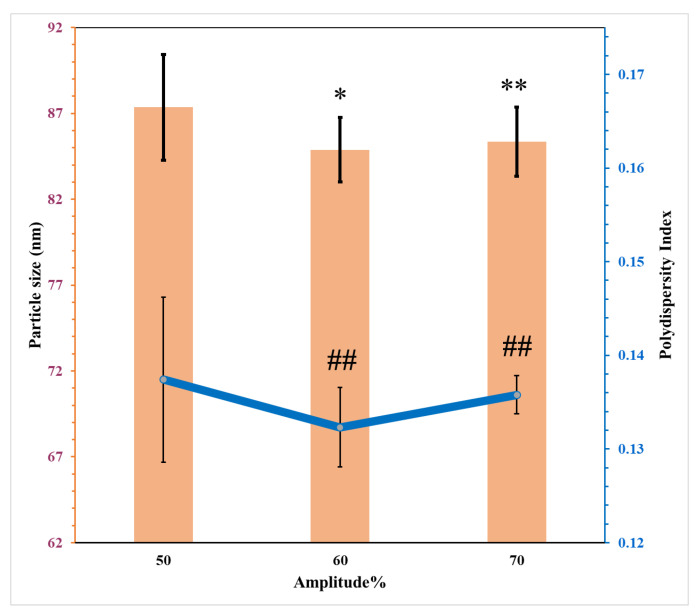
The effects of ultrasonic amplitude percent on the particle size and PDI of nanoemulsions (mean ± SD, n = 3). * significant compared to particle size of nanoemulsion with ultrasonic amplitude of 50%; ** not significant compared to particle size of nanoemulsion with ultrasonic amplitude of 50%; ## not significant compared to PDI of nanoemulsion with ultrasonic amplitude of 50%.

The influence of ultrasonication amplitude on the particle size and PDI was investigated using a constant time for homogenisation and ultrasonication, 10 and 15 min, respectively. The smallest particle size with minimum PDI was obtained when the amplitude of ultrasonication applied was 60%. The increase in the ultrasonic amplitude from 60% to 70% impacted both the particle size and the PDI value as shown in Figure 10. This can be attributed to the increase in irradiation power which increases the dispersed phase fractions for both *o*/*w* and *w*/*o* emulsion system. This increase in the irradiation power increases the pressure amplitude generated by ultrasonication which expands the extent of cavitation resulting in interfacial instability. This process leads to increased breakup of the liquid thread and eventually increases formation of the number of droplets [27,47]. In addition, the increase in the irradiation power results in elevation of temperature and this affects the droplet size as discussed previously. This phenomenon is described as Sover processing which may be a result of increased coalescence of emulsion droplets at higher shear rates [46,48].

### 3.6. Physicochemical Characterisation of Nanoemulsion Formulation

#### 3.6.1. Measurement of the Particle Size and Polydispersity Index (PDI) 

**Figure 11:**
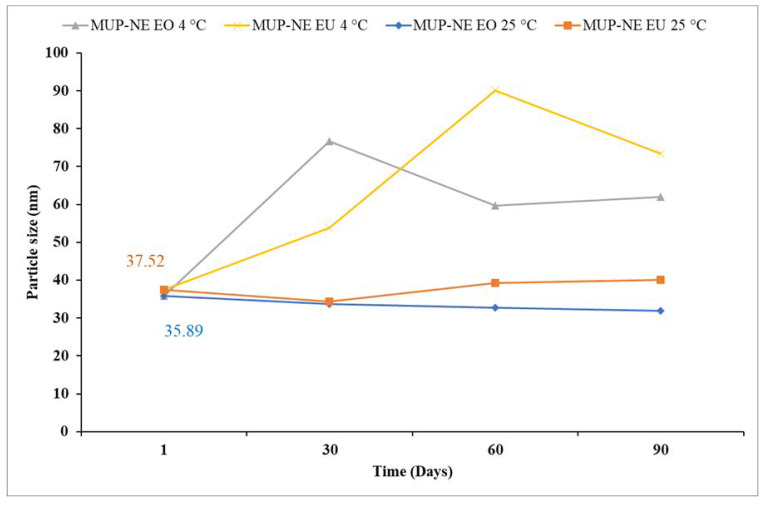
Particle size of MUP-NE EO and MUP-NE EU at 4 and 25 °C over 3 months (Mean ± SD, n = 3).

**Figure 12:**
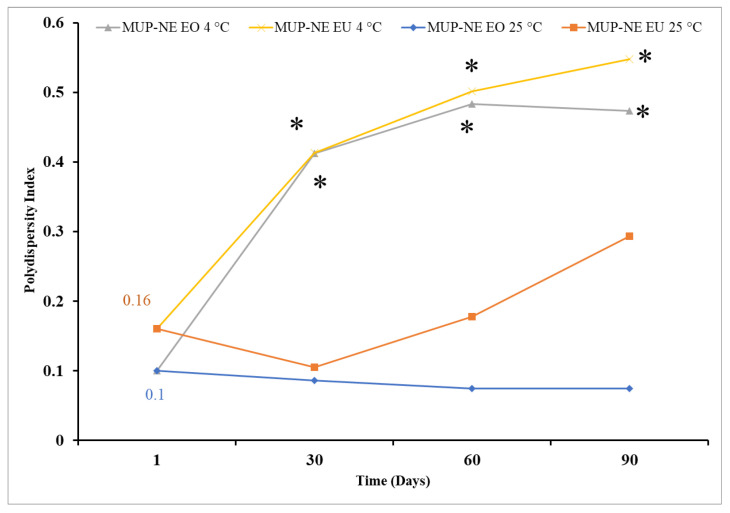
Polydispersity index of MUP-NE EO and MUP-NE EU at 4 and 25 °C over 3 months (mean ± SD, n = 3). * indicates a significant compared to PDI of the same nanoemulsion at 25 °C on the same day.

EO-based nanoemulsions had a smaller particle size with narrower PDI than EU-based nanoemulsions as shown in Figure 11 and Figure 12. Particle sizes for formulated nanoemulsions ranged between 35 and 38 nm. This size range is more likely to be absorbed through aqueous channels (aquaporins) of the skin. Moreover, these particles have the ability to penetrate the skin through transfollicular routes [49]. Figure 11 summarizes the changes in the particle size of nanoemulsions formulated using two different essential oils when stored for 3 months at 4 and 25 °C. 

Statistically, the storage temperature (4 °C and 25 °C) was shown to have a significant impact on the particle size within the nanoemulsion formulations based on EO and EU with a *p* value of less than 0.05. This might be attributed to the similarity in the chemical composition of EO with EU. The current study found the that EU constitutes 73–91% of EO composition which is in agreement with other references such as British Pharmacopoeia [50]. 

On the other hand, a change in the essential oil in the nanoemulsion formulation does not show a statistically significant change in the particle size and PDI of the nanoemulsion over 3 months at 4 °C. This was also shown the case for EO nanoemulsion stored at 25 °C. 

EO-based nanoemulsions had smaller PDI compared to EU-based nanoemulsion at 25 °C as shown in Figure 9. In addition, the statistical analysis was showed no significant change in the PDI of nanoemulsion at 4 °C regardless the type of essential oil used in the formulation. This effect was also evident at 25 °C for EO-based formulations. However, there were significant changes in the PDI of the nanoemulsion based on EU at 25 °C. On the other hand, the change in the storage temperature also had a significant effect on the PDI for nanoemulsions formulated using EO and EU. 

With low PDI values, the probability of Ostwald ripening will be low and nanoemulsions should remain stable for a long period of time [51]. Although, this was borne out statistically, it is also important to consider the practical significance. Practically, particle size and PDI are considered crucial factors in the characterisation of nanoemulsions and any deviation in these parameters, even if it is not significant statistically, requires further investigation. Similar data were obtained using both techniques, dynamic light scattering (DLS) and NTA, as shown in Table 5.

#### 3.6.2. Determination of Zeta Potential

Zeta potential indicates the interaction between droplets within a colloidal system creating a barrier with high energy against the coalescence and flocculation of nanoemulsion [52]. Zeta potentials for MUP-NE EO and MUP-NE EU were (−3.70 ± 0.36) and (−5.57 ± 0.62), respectively, as shown in Table 6. Despite high zeta potential values often being cited as a requirement for the stability of nanoemulsion, the application of this rule is restricted to dispersions with pure electrostatic stabilisers (ionic surfactants) [53]. In this study, a non-ionic ethoxylated type surfactant (Tween 80) was used that exerts its stabilising activity by a steric repulsion mechanism with the absence of strong electrical charge, masking any charges on the oil droplets [54]. 

#### 3.6.3. Determination of the Entrapment Efficiency and Drug Loading of MUP in Nanoemulsion

Concentrations of the oil and surfactant have a significant impact on the entrapment of drug through their effect on the droplet size [55]. Although the EU comprises > 80% of EO [56], EO-based nanoemulsions had higher entrapment efficiency and drug loading than EU-based nanoemulsions (Table 7). This might be due to the higher solubility of the drug in the EO, meaning that MUP can partition into EO to a greater extent than EU. 

#### 3.6.4. Fourier Transforms Infrared Spectrometry (FTIR)

**Figure 13:**
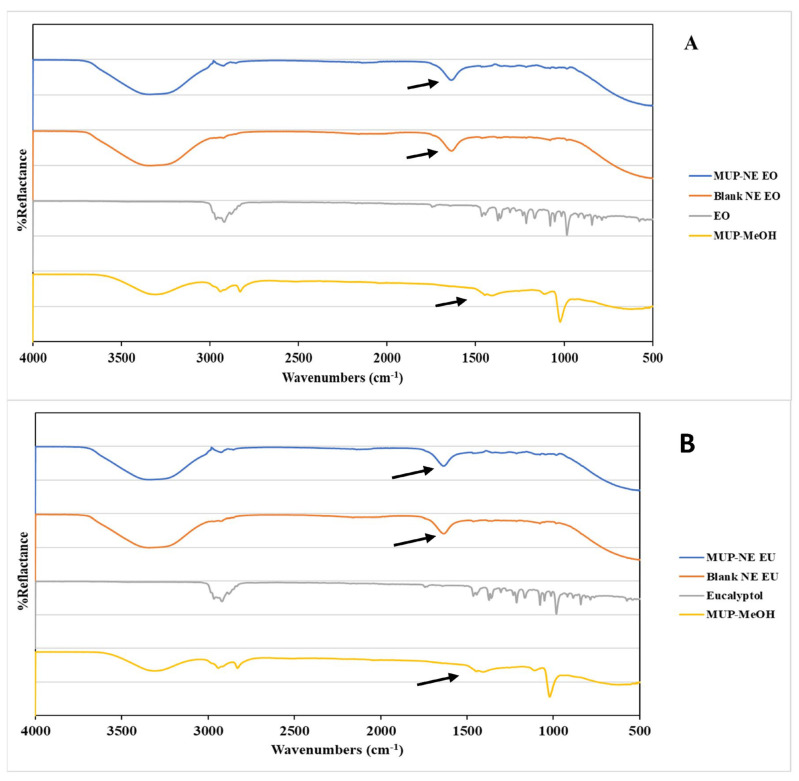
FTIR spectra for (**A**) MUP in methanol (MUP-MeOH), EO, Blank-NE EO and MUP-NE EO; (**B**) MUP-MeOH, EU, Blank-MUP-NE EU and MUP-NE EU.

The FTIR spectra show the characteristic bands of MUP in methanol, EO, EU, blank nanoemulsions comprising both oils and MUP-loaded nanoemulsion as shown in Figure 13A,B. The characteristic band for MUP at 1718.5 cm^−1^ is related to (C=O strong stretching), dual bands at 2847.6 and 2906.3 cm^−1^ related to (C−H medium stretching), and band at 1161.3 cm^−1^ is related to C−O strong stretching.

The FTIR of EO was characterised by multiple bands in the region between 2966 and 2880 cm^−1^ which corresponded to C-H strong stretching, a band at 1745 cm^−1^ related to C=O strong stretching, a band at 1664.8 cm^−1^ due to C=C weak to medium stretching of alkenes, and a band at 1445.4 cm^−1^ related to C-H medium bending of alkanes. The FTIR spectra for Blank-NE EO and MUP-NE EO had a strong broad band at 3338 cm^−1^ corresponding to O-H stretching, occurring as a result of intermolecular hydrogen bonding. The occurrence of a new band at 1633.8 cm^−1^ after nanoemulsion formulation with MUP arose due to molecular dispersion of MUP within the formulation. 

The FTIR spectrum for EU was characterised by dual bands at 2969.6 and 2922.1 cm^−1^ corresponding to C-H stretching, a band at1164.8 cm^−1^ related to C-O strong stretching of an ester, a band at 1052.9 and 1078.7 cm^−1^ due to (C-O strong stretching) of primary alcohol, and a band at 842.6 cm^−1^ corresponding to C-H bending. The FTIR spectra for Blank-NE EU and MUP-NE EO had a strong broad band at 3338 cm^−1^ corresponding to O-H stretching, occurring as a result of intermolecular hydrogen bonding. 

The occurrence of a new band at 1636.2 cm^−1^ after nanoemulsion formulation with MUP supported the development of a molecular dispersion of MUP within the formulation. In addition, the appearance of a characteristic peak between 1700 and 1500 cm^−1^ in both nanoemulsion formulations indicated by arrows in Figure 13, with and without drug, indicated an overlapping between the peak of MUP and essential oil (EO and EU). This confirmed the entrapment of MUP within the oil successfully without any evidence of significant chemical interaction between drug and oils [57]. 

### 3.7. Thermodynamic Stability Studies

Despite the thermokinetic stability of nanoemulsions, they are thermodynamically unstable. The droplet size and PDI were used as indicators to assess the stability of MUP nanoemulsion formulations.

#### 3.7.1. Long-Term Stability Studies

**Figure 14:**
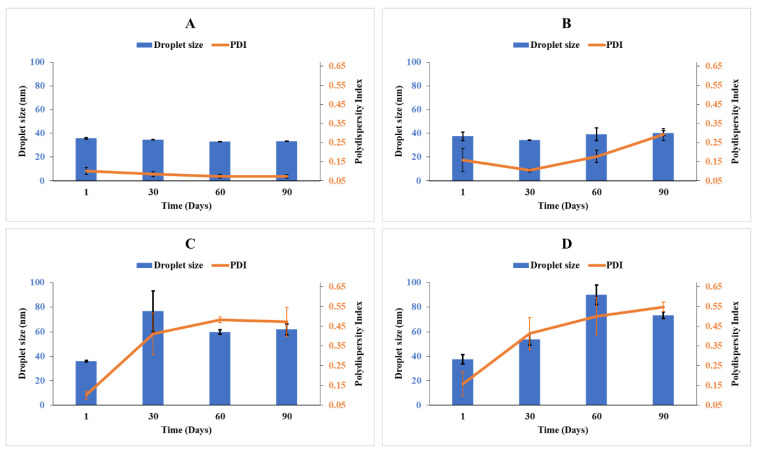
The effect of temperature on the particle size and PDI of (**A**) MUP-NE EO, (**B**) MUP-NE EU, at 25 °C, (**C**) MUP-NE EO and (**D**) MUP-NE EU at 4 °C, for 3 months (long-term stability studies) (mean ± SD, n = 3).

MUP-NE EO was more stable at 25 °C than MUP-NE EU at the same temperature over 3 months. Storage at 25 °C resulted in changes in both particle size and PDI of MUP-NE EO which was statistically not significant (*p* > 0.05) except in case of PDI after 3 months as shown in Figure 14A. The particle size and PDI of MUP-NE EU was also affected when stored at 25 °C; however, the changes are statistically significant (*p* < 0.05) as shown in Figure 14B. All nanoemulsion formulations were stable at 25 °C with slight fluctuations in size and PDI. As discussed previously, any change in the particle size and PDI should be monitored carefully even in case of statistically insignificant changes. 

Statistical analysis indicated a similar result when the nanoemulsion formulations were stored at 4 °C, with larger fluctuations in particle size and PDI. The changes in both the particle size and PDI were statistically significant (*p* < 0.05) as shown in Figure 14C,D. Despite this, the formulations underwent visible changes, becoming slightly turbid or translucent. This might be related to the increase in the PDI due to Ostwald ripening phenomena [58].

#### 3.7.2. Accelerated Stability Studies

The accelerated stability studies found that both nanoemulsion formulations passed the centrifugation and freeze–thaw cycles while instability was evident during the heating–cooling cycle. Based on the rate of separation of droplets, a centrifugation process using gravitational force was carried out and both flocculation and creaming were monitored visually. Creaming is most likely to be expected in this study because the nanoemulsion type is *o*/*w* in which the lower density portion (oil) floats on the higher density portion (water). Both nanoemulsion formulations (MUP-NE EO and MUP-NE EU) demonstrated excellent physical stability after this cycle with no creaming. This can be ascribed to the ability of nanoparticles with radii <100 nm to resist gravitational forces because of the predominant Brownian motion [59,60]. Therefore, Brownian motion of nanoparticles in both formulations (particle size ~35–38 nm), helped to keep the system stable and avoid physical instabilities indicated by flocculation at storage, sedimentation and creaming. 

**Figure 15:**
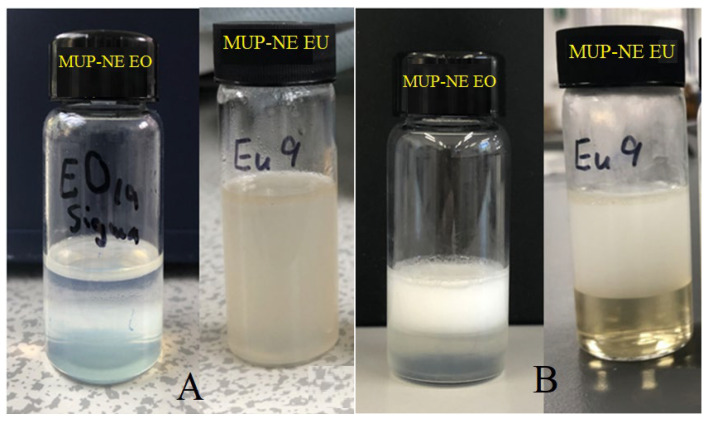
The physical appearance of MUP-NE EO and MUP-NE EU (**A**) after first heating–cooling cycle, (**B**) after fifth heating–cooling cycle.

However, neither formulation passed the heating–cooling cycle. The formulations exhibited instability when stored at 45 °C, resulting in creaming and phase separation as shown in Figure 15. These changes worsened gradually with time starting from the first heating–cooling cycle. 

Several mechanisms have been proposed to explain the cause of instability of nanoemulsions at high temperatures (45 °C in this study). The main mechanism is due to Ostwald ripening. It was proposed that higher temperatures increase the solubility of the dispersed phase in the dispersion phase. This promoted the conversion of the small droplets to smaller droplets and large droplets to larger droplets, resulting in coalescence [61]. While Galvão, et al. [62] noticed a similar issue during the storage of an *o*/*w* nanoemulsion of pepper at 37 °C, they attributed this instability to the impact of temperature on chemical reactions within the components of nanoemulsion. Boyd et al. [63] postulated an indirect effect of high temperature on the non-ionic surfactant. At 40 °C, dehydration might result in the development of a connection between the ethylene oxide and polyoxyethylene group, increasing compressive stress which presses adjacent droplets together. When the compressive stress reaches a critical point, deformation peaks resulting in a breakdown of the interfacial film surrounding the droplets and coalescence will begin.

Both formulations survived three cycles of freeze thawing without any evidence of sedimentation, creaming, coalescence and phase separation. In addition, no significant changes were detected in the particle size, PDI and zeta potential following the freeze–thaw cycles (Table 8). After multiple freeze–thaw cycles, particle size distribution was narrow for both nanoemulsion formulations. This indicated a homogenous and unimodal distribution with no significant change in the size of the droplets. Freeze–thaw testing exerts its destabilising effect by the formation of ice which squeezes the oil droplets in the remaining space of the liquid phase bringing them very close to each other, i.e., becoming aggregated. This proximity results in drainage of the water film, separating the oil droplets leading to the surfactant monolayers forming bilayer films. If the droplets have repulsive forces that cannot prevent the droplets from approaching each other and film drainage, the bilayer film tears off allowing the content of droplets to flow together leading to gradual coalescence [64,65]. Despite the destabilising ability of freeze thawing, both nanoemulsion formulations showed good stability, indicating a high repulsive force between droplets or elastic flexibility of the bilayer film between close droplets. 

### 3.8. In Vitro Release and Skin Permeation Studies

#### 3.8.1. In Vitro Release Study of MUP from Nanoemulsion Formulations

**Figure 16:**
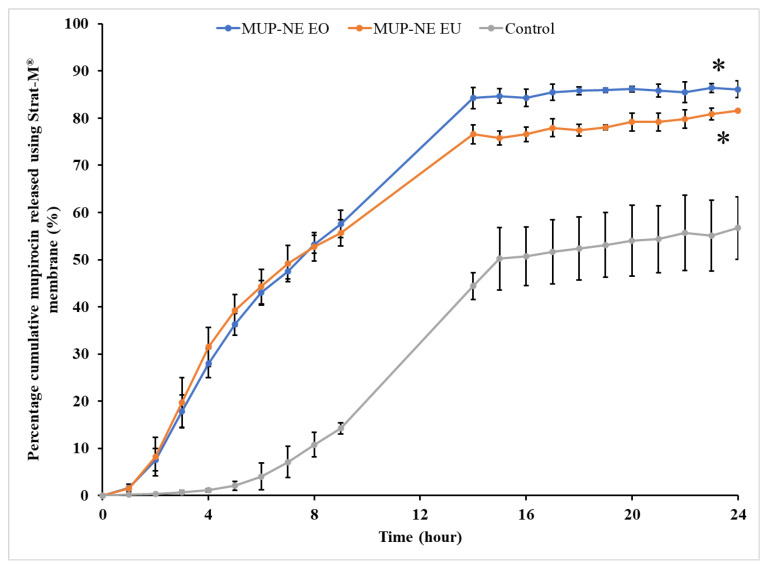
The in vitro release profile of MUP from MUP-NE EO, MUP-NE EU and control using Strat-M^®^ membrane (mean ± SD, n = 3). * indicates significant difference compared to control.

In vitro drug release studies were carried out using Strat-M^®^ membrane under sink conditions. By plotting the percentage cumulative MUP released from the MUP-NE EO, MUP-NE EU and the control cream separately versus time, drug release profiles were obtained as shown in Figure 16. The curve demonstrated a higher cumulative percentage of MUP released from MUP-NE EO as compared to MUP-NE EU and the control. The faster rate of release compared to the conventional cream formulation may be a consequence of the nanoscale droplets. However, release from all formulations plateaued after 14 h. All the nanoemulsion formulations followed zero order kinetics while the cream followed first order kinetics. Although Figure 13 appears to indicate a difference, this was not statistically significant (*p* > 0.05), although, MUP-NE EO and MUP-NE EU were different from control (*p* < 0.05). The reduction in the size of the droplets leads to an increase in the surface area of droplets which contributes to the higher solubilisation and release of drug in nanoemulsions [66]. In addition, the inclusion of essential oils such as EO and EU is also likely to impact release. Rajan and Vasudevan [67] attributed the improvement in the release of ketoconazole from transferosomal gel to the incorporation of EO in formulation, specifically EU in its composition. This has also been reported by Higgins et al. [56] and Eidv et al. [68] and is shown in the current study. 

#### 3.8.2. In Vitro Skin Permeation Study of MUP from Nanoemulsion Formulation

**Figure 17:**
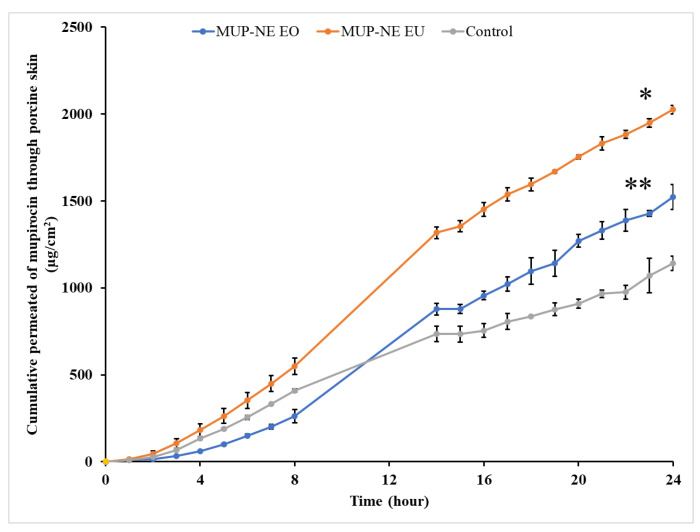
In vitro skin permeation study of nanoemulsion formulations MUP-NE EO, MUP-NE EU and control (mean ± SD, n = 3). * indicates significant difference compared to control; ** indicates no significant difference to control.

Lag time (t_lag_), steady state flux (*J*_ss_), permeability coefficient (K*_p_*) and cumulative drug permeation over 24 h (*J*_max_ or Q_24_) are shown in Table 9. MUP permeated the skin better from all nanoemulsion formulations in this study than the control. The permeation study indicated higher permeation of MUP from MUP-NE EU than MUP-NE EO as shown in Figure 17. Nanoemulsions can dissolve lipophilic drugs resulting in the improvement of the penetration through the skin layer. Many studies have reported improved permeation from nanoemulsion formulations compared to microemulsions and liposomes [69].

The direct release of drug from the nanodroplets to the stratum corneum without the need for fusion to the stratum corneum might accelerate the permeation process. This was also reported by Iskandar and Karsono [70] who compared the permeation of vitamin E from a nanoemulsion spray with a cream. The impact of particle size on the improvement in the permeation of a drug through the skin also has a significant role in the effectiveness of the formulation. MUP-NE EU showed a slightly different release and permeation from MUP-NE EO, thus the apparent increase in permeation of MUP might be affected by the nature of the formulation and may be considered as a secondary reason for this improvement in permeation. The study indicated a clear difference between MUP-NE EO and the control. This is a reason to consider the composition of each essential oil used and the predominant mechanism of penetration enhancement.

Many previous studies have reported the action of EO and EU as permeation enhancers. El-Nabarawi et al. [71] attributed the enhancement effect of EO on the permeation of sumatriptan to the modification of the nature of the stratum corneum lipids. In addition, EO was reported by Akhlaq et al. [72] to increase the skin permeability of pioglitazone through increasing the diffusivity and intermolecular space. EO increased the penetration of triclosan nanoemulsion because of its content of EU which binds in large quantities with stratum corneum lipids [73]. Although EU constitutes the main terpene in the chemical composition of EO, EO consists of a mixture of various terpene-type essential oils such as α-pinene, p-cymene and β-cymene [74]. 

Despite the higher content of EU in EO, the MUP-NE EO demonstrated lower permeability through the skin than MUP-NE EU. This indicated that EU is not the only factor in promoting permeation enhancement through the skin. The reports on the efficacy of EU as a penetration enhancer are mixed. Williams and Barry [75] found that the permeation enhancement effect of EU for lipophilic drugs is less than for hydrophilic drugs. This was attributed to its composition as oxygen-containing terpene. Despite this, EU successfully enhances the penetration of various drugs and possesses an ability to increase permeation synergistically with different substances such as surfactants and cosurfactants [76]. Subongkot et al. [77] concluded that EU increased the permeation of NaFl from ultradeformable liposomes at a rate greater than formulations without EU. 

The current study highlights the effect of EU in permeation enhancement through using it in the formulation of nanoemulsions. Although there is a clear effect in enhancing the permeation of MUP when used individually, its effect is not fully understood when combined with other essential oils artificially or naturally. Despite the high concentration of EU (80%) within EO, it works differently from EU. This might be attributed to several reasons such as the presence of plant essential oils at different thermodynamic activity levels [78]. In addition, some phytoconstitiuents within EO have different physicochemical characteristics which may hinder the transdermal permeation [79]. In addition, it is not clear which terpene or group of terpenes has the greatest effectiveness in permeation enhancement of MUP [80]. 

**Figure 18:**
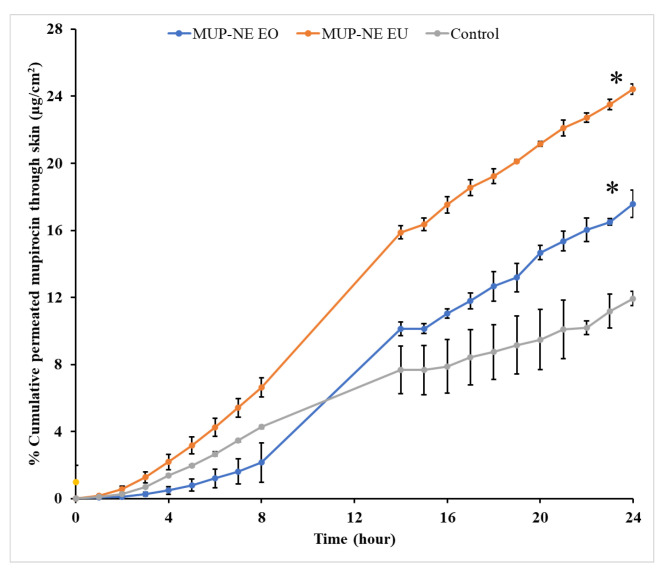
In vitro skin permeation study of nanoemulsion formulations MUP-NE EO, MUP-NE EU and control (mean ± SD, n = 3). * indicates significant difference compared to control.

Figure 18 shows a typical profile for the permeation of an infinite dose. Based on these curves, the permeation profiles such as t_lag_ and *J*_ss_ were calculated and used for the estimation of other profiles as shown in Table 9. 

The control cream results in 11.9% of percentage cumulative permeation as compared to 17.58 and 24.43% produced by MUP-NE EO and MUP-NE EU, respectively. In general, the lower percentage cumulative permeation indicates the higher deposition of drug from formulations. However, the data shown in Figure 13 refer to the highest release of MUP from MUP-NE EO (86.13%) as compared to MUP-NE EU (81.53%) and control (56.72%). Therefore, the percentage of MUP deposited from nanoemulsion formulations would be greater than the current cream formulation. Hence, the ability of nanoemulsion formulations to retain drug in the skin is greater than the control. These findings confirm the promising potential of nanoemulsion formulations for the targeting of topical infections compared to the marketed cream. 

#### 3.8.3. Quantification of MUP in Skin Using Differential Stripping Techniques

**Figure 19:**
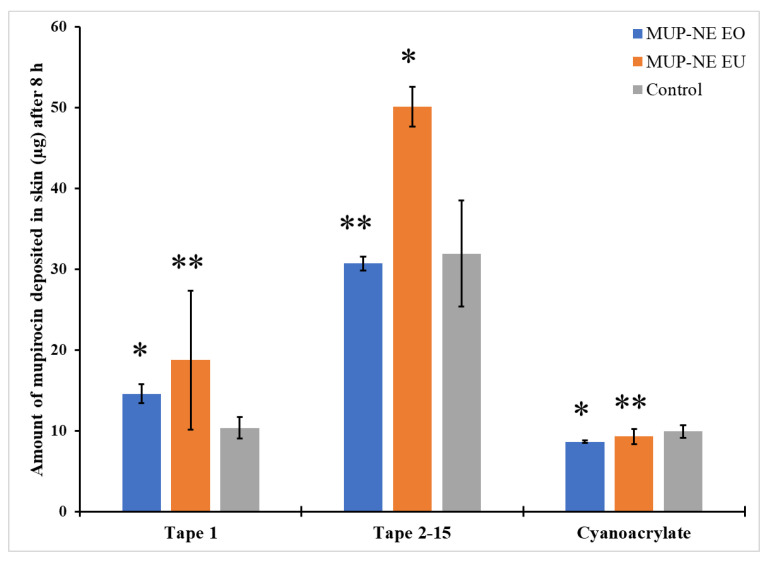
The amount of MUP penetrated the upper part of skin from MUP-NE EO, MUP-NE EU and control after 8 h (mean ± SD, n = 3). * indicates significant difference compared to control; ** indicates no significant difference to control.

**Figure 20:**
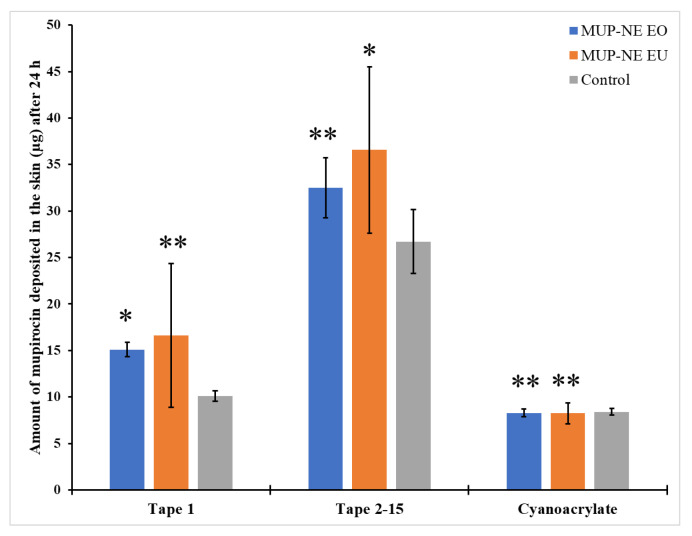
The amount of MUP penetrating the upper part of skin from MUP-NE EO, MUP-NE EU and control after 24 h (mean ± SD, n = 3). * indicates significant difference compared to control; ** indicates no significant difference to control.

The ability of the formulation to penetrate the skin and the impact of the application time are shown in Figure 19 and Figure 20. After 8 h, MUP-NE EU resulted in the highest penetration and retention of drug in the skin while the penetration was lower at 24 h. In other words, deposition of MUP is enhanced with MUP-NE EU as compared to other nanoemulsions and control. EU increases the solubility of drugs in the nanoemulsion and according to Fick’s first law, the flux is directly proportional to the concentration of drug in the vehicle. Additionally, EU helps the drug to partition into stratum corneum lipids resulting in producing a higher concentration of drug in the upper part of the skin as represented in Figure 16. This increase in the concentration gradient acts as a driving force for the transdermal penetration of drug. This agreed with the findings of Liu et al. [81]. However, no significant changes were evident regarding the penetration of drug from MUP-NE EO and the control cream between 8 and 24 h following application. In addition, the study found statistically non-significant changes in the penetrated amount of drug from all the formulations in the uppermost part of the skin and hair follicles after 8 and 24 h following application. 

**Figure 21:**
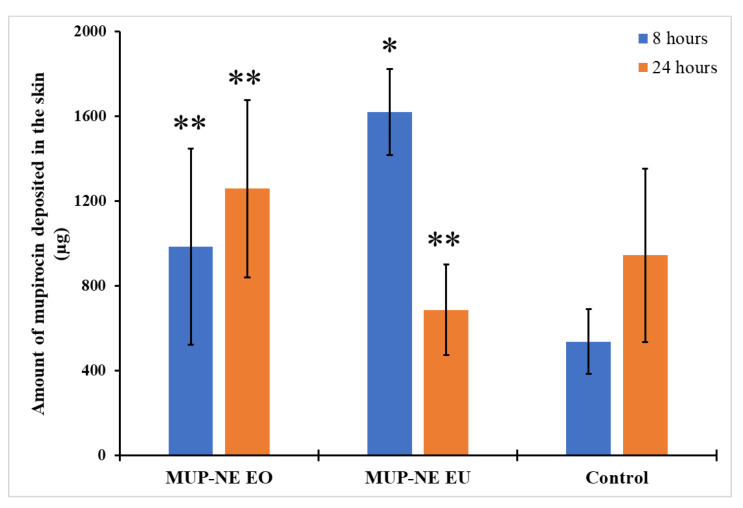
The amount of MUP deposited in the deeper part of skin from MUP-NE EO, MUP-NE EU and control (mean ± SD, n = 3) after 8 and 24 h. * indicates significant difference compared to control; ** indicates no significant difference to control.

The current study also found penetration and deposition of the drug in deeper skin after 8 h of application was in the following order: MUP-NE EU > MUP-NE EO > cream (Figure 21). However, after 24 h, the deposited amount of drug from MUP-NE EU decreased, while the amount of drug deposited from MUP-NE EO and the control was increased by 28% and 75%, respectively. The interaction between the vehicle (essential oil) and the skin result in the disruption of the stratum corneum lipid barrier and increase fluidisation of the stratum corneum. The ability to exert this effect depends on the type of essential oil used [78]. Cornwell et al. [82] and Cal et al. [83,84] reported the ability of EU (main constituent of EO) to bind in the stratum corneum and hence enhance lipophilic drug penetration by increasing the partition coefficient. In addition, they showed that EU tends to deposit within the skin more than readily than permeate through it. Fluidisation of the stratum corneum lipids produces less resistance against the penetration of MUP into the stratum corneum, so MUP-NE EU faced the least resistance for penetration as compared with MUP-NE EO and the control regardless of application time. Moreover, it may be due to the greater ability of EU to enhance the permeation of a drug [85]. In addition, the direct transport of drug from nanoemulsion formulations and distribution due to its small droplet size and large surface area might also result in the deposition of the drug in the upper part of the skin [70]. This increases the concentration gradient which enhances the permeation of the drug at the expense of deposition to the skin after a few hours [81]. This might show the reduction in MUP deposition from MUP-NE EU and show an increase in it from MUP-NE EO and the control after 24 h, as represented in Figure 21. 

Additionally, the comparatively low viscosity of nanoemulsion formulations is more likely to facilitate penetration and partition of drug into skin compared to a conventional cream. Moreover, the solubility of drugs in the essential oil may impact the rate of drug release from the nanoemulsion formulations and deposition into the skin [70].

Molecules with higher solubility in lipid will penetrate faster into cells. Eman Abd et al. [17] suggested a mathematical model to estimate and predict the effect of the solubility of drugs in the stratum corneum as shown in Equation (6).

(6) $$K_{p}=\frac{J_{max}}{S_{SC}}$$

where: 

*K_p_*: permeability coefficient; 

*J_max_*: the maximum flux; 

*S_sc_*: the solubility of drug in stratum corneum.

As the droplet sizes of nanoemulsions comprising EO and EU are similar (MUP-NE EO = 35.89 ± 0.68 and MUP-NE EU = 37.52 ± 3.65 nm), the solubility of drugs in the stratum corneum must play an important role in determining the penetration and permeation especially when the thermodynamic activity is constant. In addition, the sebaceous lipid provides a suitable environment for the nanoemulsion to be deposit in hair follicles [86]. However, the situation with control cream formulation is different. Although it has a high permeability coefficient, it did not permeate as effectively. This is likely due to the larger droplet size which might result in the deposition within the hair follicles because of its tendency to pass through the transappendageal pathway. 

## 4. Conclusions

In summary, an optimised nanoemulsion formulation was successfully developed for topical drug delivery for MUP as an antibacterial drug, EO and EU as oil phase and T80:S80 mixture as a surfactant:cosurfactant mixture at a ratio of 2:1. A stable nanoemulsion was obtained at 25 °C with mean droplet size of less than 38 nm and narrow PDI of less than 0.17 maintained over 3 months. MUP-NE EU showed no significant difference in topical permeability as compared to MUP-NE EO. However, it has significantly higher permeability than the control, a marketed cream. In addition, MUP-NE EU resulted in greater deposition of MUP in the skin than MUP-NE EO and the control following 8 h of application, while MUP-NE EO demonstrated superiority in drug deposition in skin following 24 h of application as compared to MUP-NE EU and the control. These findings highlighted the possibility for the development of nanoemulsions based on different essential oils for the acute or prophylactic management of skin infection. This requires further investigation of the concept through studying the effect of these essential oils using different model drugs. In addition, an in vivo skin uptake study would be useful at later stages to determine the pharmacokinetic profile of the drugs. 

## Figures and Tables

**Table 1:** Composition of the nanoemulsion with/without MUP.

	Quantity (% *w*/*w*)			
Formulation Code	Blank-NE EO	Blank-NE EU	MUP-NE EO	MUP-NE EU
Aqueous phase	90	90	90	90
T80	5	5	5	5
Deionised water	85	85	84.6	84.6
Oil phase	10	10	10	10
S80	2.5	2.5	2.5	2.5
EO	7.5		7.5	
EU		7.5		7.5
MUP			0.4	0.4

**Table 2:** Independent and dependent variables created based on CCD for nanoemulsion formulation.

Independent Variables		Level			Dependent Variables		Aim
		Low (−1)	Medium (0)	High (+1)			
*X*_1_	Homogenisation time (min)	10	7.5	5	*Y*_1_	Particle size (nm)	Reduction
*X*_2_	Ultrasonication time (min)	20	15	10	*Y*_2_	PDI	Reduction
*X*_3_	Amplitude (%)	70	60	50			

**Table 3:** Experimental plan based on central composite design.

Run	Homogenisation Time (min)	Ultrasonication Time (min)	Amplitude (%)
1	5	10	50
2	10	10	50
3	5	20	50
4	10	20	50
5	5	10	70
6	10	10	70
7	5	20	70
8	10	20	70
9	5	15	60
10	10	15	60
11	7.5	10	60
12	7.5	20	60
13	7.5	15	50
14	7.5	15	70
15	7.5	15	60
16	7.5	15	60
17	7.5	15	60

**Table 4:** The solubility of MUP (mean ± SD, n = 3).

Components	Solubility of MUP (mg/mL)
Eucalyptus oil (EO)	21.00 ± 0.17
Eucalyptol (EU)	7.94 ± 0.25
Methanol (MeOH)	189.67 ± 0.52
Absolute ethanol (EtOH)	179.24 ± 0.34
Phosphate buffer saline (PBS)	3.39 ± 0.11
MeOH-PBS (1:9)	3.51 ± 0.13
MeOH-PBS (3:7)	10.17 ± 0.10
MeOH-PBS (5:5)	39.51 ± 0.27
Deionised water	1.64 ± 0.23
Acetonitrile	32.86 ± 0.21
T80 in water (4.91% *w*/*w*)	2.24 ± 0.06
S80 in EO (4.44% *w*/*w*)	23.05 ± 0.23
S80 in EU (4.89% *w*/*w*)	22.57 ± 0.08
T80: S80: EO (4.60% *w*/*w*)	26.13 ± 0.02
T80: S80: EU (5.23% *w*/*w*)	25.89 ± 0.22

**Table 5:** Average particle size and PDI determined by DLS, and cumulative data (D_10_, D_50_, D_90_) determined by NTA for MUP-NE EO and MUP-NE EU (Mean ± SD, n = 3).

Formulations	DLS		NTA				
	Average Particle Size (nm)	PDI	Cumulative Data			Mean Size (nm)	Span
			D_10_	D_50_	D_90_		
MUP-NE EO	35.89 ± 0.68	0.10 ± 0.02	19	28	44	31.0 ± 1.0	0.89
MUP-NE EU	37.52 ± 3.65	0.16 ± 0.06	21	29	47	33.3 ± 1.5	0.90

**Table 6:** The S_mix_ and oil concentration with physicochemical characteristics of both nanoemulsion formulations (MUP-NE EO and MUP-NE EU) (mean ± SD, n = 3).

Formulations	S_mix_	Oil Concentration	Zeta Potential (mV)	pH	Viscosity (cP)
MUP-NE EO	7.50%	7.50%	−3.7 ± 0.36	3.91 ± 0.03	38.15 ± 0.87
MUP-NE EU	7.50%	7.50%	−5.57 ± 0.62	4.13 ± 0.05	25.85 ± 6.22

**Table 7:** Drug incorporation and entrapment efficiency for MUP-NE EO and MUP-NE EU, (mean ± SD, n = 3).

Formulation	Amount of Drug in Nanoemulsion (mg)	Entrapment Efficiency (EE%)
MUP-NE EO	316.57 ± 48.58	79.14 ± 12.15
MUP-NE EU	276.63 ± 8.08	69.15 ± 2.02

**Table 8:** The particle size, PDI and zeta potential of samples passing freeze–thaw cycle (mean ± SD, n = 3).

Formulation	Particle Size (nm)	PDI	Zeta Potential (mV)
MUP-NE EO	33.21 ± 0.21	0.09 ± 0.01	−4.53 ± 0.88
MUP-NE EU	34.54 ± 0.27	0.10 ± 0.01	−3.79 ± 0.89

**Table 9:** The permeation profile of MUP-NE EO, MUP-NE EU and Bactroban^®^ cream using porcine skin (mean ± SD, n = 3).

Parameters	MUP-NE EO	MUP-NE EU	Control
t_lag_ (h)	2.93	1.7	1.86
*J*_max_ (µg/cm^2^)	1522.72 ± 70.87	2025.1 ± 25.42	1141.61 ± 40.45
*J_ss_* (µg/cm^2^/h)	68.35 ± 2.87	72.31 ± 2.46	39.32 ± 4.27
K*_p_* (×10^−4^ cm/h)	87.22 ± 2.97	66 ± 19.1	40.5 ± 4.4
Enhancement ratio (ER)	1.33	1.77	1

## Data Availability

The data can be shared up on request.

## Supplementary Materials

The following supporting information can be downloaded at: https://www.mdpi.com/article/10.3390/pharmaceutics15020378/s1, Table S1: The obtained response (actual and predicted) for nanoemulsion formulations of CCD trials; Table S2: ANOVA data for droplet size of nanoemulsions; Table S3: ANOVA data for PDI of nanoemulsions.
