# Supplementary material for: Development of Nanoemulsions for Topical Application of Mupirocin
Source: Pharmaceutics. 2023 Jan 22;15(2):378. doi: 10.3390/pharmaceutics15020378 (PMC9960479; doi:10.3390/pharmaceutics15020378)
Supplement: Supplementary file 1 [file pharmaceutics-15-00378-s001.zip › pharmaceutics-2134612-supplementary.pdf]

Supplementary materials

Table S1 The obtained response (actual and predicted) for nanoemulsion formulations of CCD trials.

Run	Independent variables			Dependant variables			
	X_1: Homogenisation time (min)	X_2: Ultrasonication time (min)	X_3: Amplitude (%)	Y_1: Size (nm)		Y_2: PDI	
				Actual	Predicted	Actual	Predicted
1	7.5	15	60	86.23	85.75	0.137	0.136
2	5	10	50	91.69	88.39	0.150	0.148
3	10	10	50	87.75	88.39	0.141	0.142
4	5	20	50	88.41	85.10	0.127	0.127
5	10	20	50	83.81	85.10	0.137	0.130
6	5	10	70	88.95	86.40	0.134	0.139
7	10	10	70	84.47	86.40	0.139	0.137
8	5	20	70	84.62	83.11	0.132	0.129
9	7.5	15	60	84.27	85.75	0.126	0.136
10	10	20	70	84.47	83.11	0.137	0.137
11	5	15	60	85.16	85.75	0.136	0.136
12	10	15	60	82.60	85.75	0.129	0.136
13	7.5	10	60	88.20	87.39	0.152	0.141
14	7.5	20	60	83.30	84.10	0.132	0.131
15	7.5	15	50	85.10	86.74	0.132	0.137
16	7.5	15	70	84.30	84.75	0.137	0.135
17	7.5	15	60	84.42	85.75	0.134	0.136

Table S2 ANOVA data for droplet size of nanoemulsions.

Source	DF	Adjusted Sum of Square	Adjusted Mean Square	*F*-Value	*p*-Value
Model	9	83.7997	9.3111	6.43	0.011
Linear	3	61.7212	20.5737	14.22	0.002
X_1: Homogenisation time (min)	1	24.7506	24.7506	17.1	0.004
X_2: Ultrasonication time (min)	1	27.0789	27.0789	18.71	0.003
X_3: Amplitude (%)	1	9.8916	9.8916	6.84	0.035
Square	3	17.4404	5.8135	4.02	0.059
X_1^2	1	0.0607	0.0607	0.04	0.844
X_2^2	1	7.9066	7.9066	5.46	0.052
X_3^2	1	1.201	1.201	0.83	0.393
2-Way Interaction	3	4.6382	1.5461	1.07	0.422
X_1X_2	1	1.6845	1.6845	1.16	0.316
X_1X_3	1	1.9185	1.9185	1.33	0.287
X_2X_3	1	1.0351	1.0351	0.72	0.426
Error	7	10.1298	1.4471		
Lack-of-Fit	5	7.7544	1.5509	1.31	0.487
Pure Error	2	2.3754	1.1877		
Total	16	93.9295			

Table S3 ANOVA data for PDI of nanoemulsions.

Source	DF	Adjusted Sum of Square	Adjusted Mean Square	*F*-Value	*p*-Value
Model	6	0.000396	0.000066	1.75	0.208
Linear	3	0.000269	0.00009	2.37	0.132
X_1: Homogenisation time (min)	1	0.000001	0.000001	0.01	0.907
X_2: Ultrasonication time (min)	1	0.000264	0.000264	6.98	0.025
X_3: Amplitude (%)	1	0.000005	0.000005	0.13	0.726
2-Way Interaction	3	0.000127	0.000042	1.12	0.387
X_1X_2	1	0.000045	0.000045	1.19	0.3
X_1X_3	1	0.000012	0.000012	0.31	0.59
X_2X_3	1	0.00007	0.00007	1.85	0.203
Error	10	0.000378	0.000038		
Lack-of-Fit	8	0.000314	0.000039	1.23	0.522
Pure Error	2	0.000064	0.000032		
Total	16	0.000774			
